# OPTimal IMAging strategy in patients suspected of non-traumatic pulmonary disease at the emergency department: chest X-ray or ultra-low-dose chest CT (OPTIMACT) trial—statistical analysis plan

**DOI:** 10.1186/s13063-020-04343-w

**Published:** 2020-05-14

**Authors:** Maadrika M. N. P. Kanglie, Shandra Bipat, Inge A. H. van den Berk, Tjitske S. R. van Engelen, Marcel G. W. Dijkgraaf, Jan M. Prins, Jaap Stoker, Patrick M. M. Bossuyt

**Affiliations:** 1 Department of Radiology and Nuclear Medicine, Amsterdam UMC, location AMC, University of Amsterdam, P.O. Box 22660, 1100 DD Amsterdam, the Netherlands; 2 Center of Experimental and Molecular Medicine, Amsterdam UMC, University of Amsterdam, P.O. Box 22660, 1100 DD Amsterdam, the Netherlands; 3 Department of Clinical Epidemiology, Biostatics and Bioinformatics, Amsterdam UMC, location AMC, University of Amsterdam, P.O. Box 22660, 1100 DD Amsterdam, the Netherlands; 4 Department of Internal Medicine, Amsterdam UMC, University of Amsterdam, P.O. Box 22660, 1100 DD Amsterdam, the Netherlands

**Keywords:** Ultra-low-dose chest CT, ULD chest CT, Microdose chest CT, Chest X-ray, Non-traumatic pulmonary disease, Non-traumatic chest disease, Pulmonary disease, Emergency department, Statistical analysis plan

## Abstract

**Background:**

A chest X-ray is a standard imaging procedure in the diagnostic work-up of patients suspected of having non-traumatic pulmonary disease. Compared to a chest X-ray, an ultra-low-dose (ULD) chest computed tomography (CT) scan provides substantially more detailed information on pulmonary conditions. To what extent this translates into an improvement in patient outcomes and health care efficiency is yet unknown. The OPTimal IMAging strategy in patients suspected of non-traumatic pulmonary disease at the emergency department: chest X-ray or ultra-low-dose chest CT (OPTIMACT) study is a multicenter, pragmatic, non-inferiority randomized controlled trial designed to evaluate replacement of chest X-ray by ULD chest CT in the diagnostic work-up of such patients, in terms of patient-related health outcomes and costs. During randomly assigned periods of 1 calendar month, either conventional chest X-ray or ULD chest CT scan was used as the imaging strategy. This paper presents in detail the statistical analysis plan of the OPTIMACT trial, developed prior to data analysis.

**Methods/results:**

Functional health at 28 days is the primary clinical outcome. Functional health at 28 days is measured by the physical component summary scale of the Short Form (SF)-12 questionnaire version 1. Secondary outcomes are mental health (mental component summary scale of the SF-12), length of hospital stay, mortality within 28 days, quality-adjusted life year equivalent during the first 28 days (derived from the EuroQol five-dimension, five-level instrument), correct diagnoses at emergency department discharge as compared to the final post hoc diagnosis at day 28, number of patients in follow-up because of incidental findings on chest X-ray or ULD chest CT, and health care costs.

**Conclusions:**

After this pragmatic trial we will have precise estimates of the effectiveness of replacing chest X-ray with ULD chest CT in terms of patient-related health outcomes and costs.

**Trial registration:**

Netherlands National Trial Register: NTR6163. Registered on 6 December 2016.

## Introduction

In patients suspected of having non-traumatic pulmonary disease, a chest X-ray is a standard diagnostic procedure. Being a two-dimensional projection technique, the chest X-ray has limitations [1–8]. As a cross-sectional three-dimensional imaging technique, a computed tomography (CT) scan better highlights chest anatomy and pathology, at the expense of a higher radiation dose [9–11]. However, recently iterative reconstruction has been introduced, allowing the introduction of CT scanners with intrinsically lower radiation exposure for any application [12–14].

Compared to chest X-ray, ultra-low-dose chest CT (ULD chest CT) provides substantially more detailed information on non-traumatic pulmonary conditions, with a radiation dose in the order of that of the chest X-ray (0.1 vs. 0.05 mSv), and even lower doses seem in reach. To what extent this translates into an improvement in patient outcomes and health care efficiency is yet unknown.

The OPTimal IMAging strategy in patients suspected of non-traumatic pulmonary disease at the emergency department: chest X-ray or CT (OPTIMACT) trial is designed to evaluate the effects of replacing chest X-ray by ULD chest CT in daily practice, in terms of patient-related health outcomes and costs, in the diagnostic work-up of patients suspected of non-traumatic pulmonary disease at the emergency department (ED). The study protocol was previously published [15]; it is currently at version 3.2, dated 4 may 2018, revision version 1. Here we present the statistical analysis plan (SAP) for the outcome data, developed by the investigators prior to data analysis.

### Summary of study protocol

OPTIMACT is a multicenter, pragmatic, non-inferiority randomized controlled trial (RCT) comparing ULD chest CT to chest X-ray in consecutive patients suspected of having non-traumatic pulmonary disease presenting at the ED. For the complete study protocol, we refer to a previous publication [15].

During randomly assigned periods of 1 calendar month, either conventional chest X-ray or ULD chest CT scan was used as the imaging strategy. The two strategies were randomly allocated using computer-generated blocks of 2 months. Patients were enrolled in two participating Dutch hospitals, one university hospital (Amsterdam University Medical Centers [Amsterdam UMC], location Academic Medical Center [AMC], Amsterdam) and one large teaching hospital (Spaarne Gasthuis [SG]), with two locations (Haarlem and Hoofddorp).

Eligible were consecutive adult patients presenting at the ED with suspected non-traumatic pulmonary disease, either self-referred or referred by a general practitioner or their treating physician at the hospital. Inclusion criteria were age ≥ 18 years and presentation at the ED with a suspicion of non-traumatic pulmonary disease, in people for whom chest X-ray was required for work-up by the attending physician. Excluded were patients unable to undergo a chest X-ray or ULD chest CT (e.g., those not able to lay in a supine position), incapacitated patients, pregnant ones, those with a life expectancy of less than 1 month, patients with anticipated barriers to complete follow-up data collection, and earlier participants in this RCT. The Medical Ethics Committee of the Amsterdam UMC approved the study protocol, and written informed consent (IC) was provided by all study participants.

**Fig. 1:**
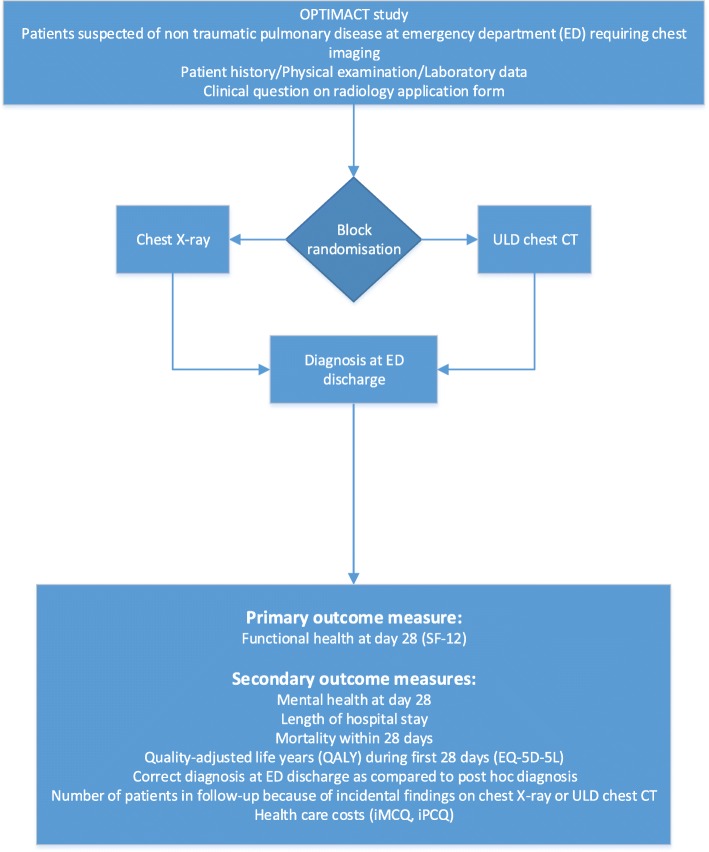
Flowchart work-up for OPTIMACT study

The initial examination by the attending physician consisted of a standardized clinical history and physical examination, including mental state. A predefined laboratory set was ordered. The attending physician evaluated this information and provided a working diagnosis and its probability on the radiology application form. The imaging method allocated to the month of presentation (chest X-ray or ULD chest CT) was performed. Structured standardized reporting of the images took place. Examinations were read or supervised by radiologists experienced in chest radiology, also outside regular office hours. The radiologist also assigned an imaging diagnosis and probability. The results of the chest X-ray or ULD chest CT were communicated directly to the attending physician, after which the attending physician formulated a final clinical diagnosis (Fig. 1).

If the clinical question was not adequately answered after obtaining the chest X-ray or ULD chest CT, standard additional imaging (e.g., chest CT with intravenous contrast medium, CT pulmonary angiography) could be performed, in concordance with regular clinical practice. Initial and subsequent treatment, including antibiotic treatment, duration of treatment, and discharge from the hospital, was at the discretion of the attending physician, if applicable according to current Dutch guidelines [16, 17].

The primary clinical outcome is functional health at day 28. Functional health at day 28 was measured by the physical component summary (PCS) scale of the Short Form (SF)-12 questionnaire version 1 (SF-12 v.1). Secondary outcomes are mental health (measured by the mental component summary [MCS] scale of the SF-12 v.1), length of hospital stay, mortality within 28 days, quality-adjusted life year (QALY) equivalent during the first 28 days (based on the EuroQol five-dimension, five-level [EQ-5D-5L] questionnaire health status scoring profiles), correct diagnosis at ED discharge as compared to the final post hoc diagnosis at day 28, number of patients in follow-up because of incidental findings on chest X-ray or ULD chest CT, and health care costs (assessed by Institute for Medical Technology Assessment [iMTA] Medical Consumption Questionnaire [iMCQ] and Productivity Cost Questionnaire [iPCQ]; both questionnaires were adapted to the study setting) [18, 19]. Study participants received the questionnaires (SF-12 v.1, EQ-5D-5L, iMCQ, and iPCQ) as a single document, either by mail or e-mail, according to the preference of the participant.

The investigational product, ULD chest CT, is a non-invasive imaging method used in standard clinical care. The CT scanners used in the trial are under intensive, regular quality control. Safety reporting will therefore be limited to events possibly related to the study procedure (chest X-ray vs. CT scan) and only for the period during which the patient is admitted to the ED. Chest X-ray and ULD chest CT results will be read by qualified personnel.

We hypothesize that introduction of ULD chest CT would reduce costs while being at least equivalent to chest X-ray regarding functional health outcome. A standard deviation (SD) of 10 on the SF-12 v.1 was anticipated [20]. The power analysis showed that, using a 0.05 significance level and 80% power to exclude a 1-point difference (margin) in the mean SF-12 v.1 physical summary scale score, with the two-sample *t* test statistic, 2400 participants were needed assuming no difference in the mean scores. This 1-point non-inferiority margin comes down to a 0.1 effect size.

Given an anticipated 63% inclusion rate (based on pilot data), the inclusion of 3810 potentially eligible patients was deemed necessary. Every month, 705 potentially eligible patients are seen in both hospitals combined. As the study started earlier at the Amsterdam UMC, location AMC, we did not aim at an equal contribution of both hospitals. Enrollment of study participants stopped at the end of the month during which the projected 2400 inclusions were reached. The last patient was included on 31 May 2018.

### Statistical analysis plan

#### Study objectives

The primary goal is to evaluate the effects of replacing chest X-ray by ULD chest CT in the diagnostic work-up of patients suspected of having non-traumatic pulmonary disease at the ED in terms of patient-related health outcomes and cost-effectiveness. The secondary goal is to evaluate whether the replacement of chest X-ray by ULD chest CT leads to more accurate diagnoses at ED discharge.

#### Overall principles

The data analysis will start after the study database is cleaned and locked. The analyses will be done by co-investigators (MMNPK, IAHvdB, TSRvE), supervised by the principal investigators (JS and JMP), epidemiologists (PMMB and SB), and a health economics expert (MGWD). The statistical programming and analysis to produce all tables and figures will use the Statistical Package for Social Sciences v. 26 (IBM Corporation, Armonk, NY, USA) or R studio (R language and environment for statistical computing, v. 2.15.1; R Foundation for Statistical Computing, Vienna, Austria; http://www.R-project.org/).

In general, descriptive statistics, such as means with SD for continuous normally distributed variables, medians and interquartile ranges for continuous skewed variables, and frequencies with percentages for categorical variables, will be used to summarize variables.

A one-sided *P* value of < 0.05 will be used to evaluate the null hypothesis of non-inferiority.

Because of the non-inferiority design, no adjustment for multiplicity is planned. No interim analysis was planned. All results and conclusions will be based on the final analysis of this trial.

Diagnostic test characteristics of chest X-ray and ULD chest CT will be reported according to the Standards for Reporting of Diagnostic accuracy studies (STARD) guidelines [21].

#### Intention-to-imaging (ITI) and imaging-per-protocol (IPP) populations

**Fig. 2:**
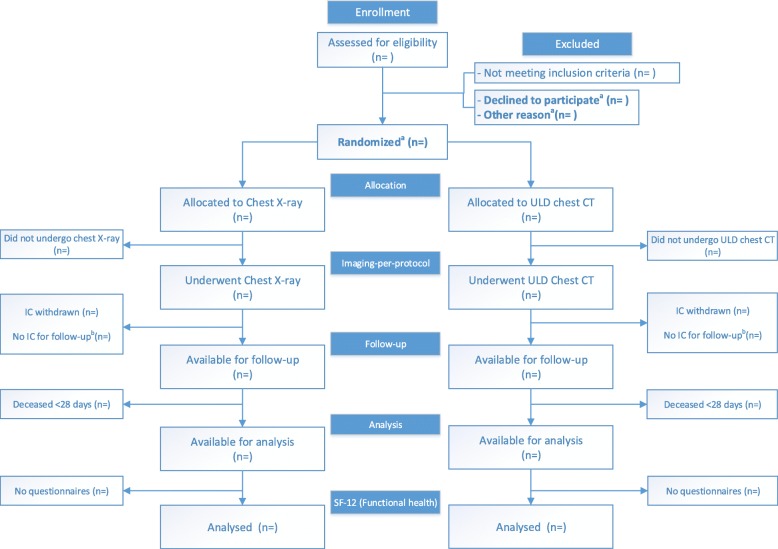
Flowchart of patients for assessment of the primary outcome SF-12 (PCS). ^a^Intention-to-imaging population. ^b^Short informed consent form signed in acute phase at the emergency department giving permission to only use imaging information for study purposes. No full informed consent form, giving permission for collection of follow-up information, was signed

The ITI population includes all patients referred for imaging who fulfill all the inclusion criteria and none of the exclusion criteria. The IPP population includes all patients who actually received the allocated imaging procedure according to the randomization scheme, thereby excluding patients who were offered another diagnostic trajectory for logistic or other reasons (e.g., CT not available, multiple patients presenting simultaneously) (Fig. 2).

#### Protocol deviations

All substantial protocol deviations will be summarized by treatment group with details of type of deviation provided. Substantial protocol deviations are as follows: the inclusion of patients despite meeting one or more exclusion criteria, and not undergoing the allocated imaging modality.

#### Handling of missing data

We will consider patients lost to follow-up if IC was withdrawn or in the presence of only a short IC form signed in the acute phase at the ED, providing permission to only use the imaging data of the ED visit (Fig. 2).

Missing primary outcome data of the remaining patients will be imputed where possible using multiple imputations, relying on a Markov chain Monte Carlo approach with fully conditional specification. We will perform separate analyses on the non-imputed dataset (primary analysis) and on the imputed datasets (sensitivity analysis).

#### List of analyses

The primary analyses will be performed in the ITI population. Baseline characteristics will be compared between the ITI and IPP populations.

#### Recruitment and retention

The flow of participants will be displayed in a Consolidated Standards of Reporting Trials (CONSORT) flow diagram (Fig. 2).

#### Baseline characteristics

The baseline characteristics of all study participants will be presented in a table stratified for imaging technique. The following variables will be reported: age, gender, comorbidity (derived from the Charlson Comorbidity Index [22]), presenting symptoms, and clinical question on radiology application form (see Tables 1 and 2).

**Table 1:** Baseline characteristics

Item	Specification	Ultra-low-dose chest CT (*N*)	Chest X-ray (*N*)
**Age, years (mean, +/−SD)**			
**Gender, F (*n*, %)**			
**Comorbidity (*n*, %)**			
Diabetes	With end organ failure^c^		
	No end organ failure^c^		
Liver disease	Chronic hepatitis		
	Cirrhosis		
Solid tumor^a^	Locally advanced		
	Metastasized		
Kidney disease	Any		
History of myocardial infarction			
Chronic cardiac failure^b^			
Cerebrovascular disease^b^			
Dementia^b^			
Peripheral vascular disease^b^			
Hemiplegia^b^			
Connective tissue disease^b^			
Leukemia^a^			
Malignant lymphoma^a^			
History of intestinal ulcer(s)^b^			
Chronic obstructive pulmonary disease			
Asthma			
Cystic fibrosis			
Interstitial lung disease			
History of thrombo-embolic disease			
Sickle cell disease			
Immunocompromised	HIV positive		
	Neutropenia		
	Chemotherapy		
	Leukemia/lymphoma		
	Organ transplant		
	Immunosuppressive medication		
	Total		

^a^Within the past 5 years, except for chronic lymphatic leukemia

^b^See Charlson Comorbidity Index for exact clarification [22]

^c^End organ failure: retinopathy, neuropathy, or nephropathy

**Table 2:** **Diagnostic baseline characteristics**

Item	Ultra-low-dose chest CT (***N***)	Chest X-ray (***N***)
**Presenting symptoms (*n*, %)**		
Cough		
Dyspnea		
Sputum		
Hemoptysis		
Thoracic pain		
Fever		
Confusion		
**Clinical question ULD chest CT/chest X-ray (*n*, %)**		
Pneumonia		
Bronchitis		
Bronchiolitis		
Congestion		
Pneumothorax		
Pleural effusion		
Atelectasis		
Emphysema		
Pulmonary tumor		
Pulmonary metastases		
Lymphoma		
Other		

#### Assessment and analysis of primary outcome

Patients’ functional health, the primary clinical outcome measure, is assessed using the PCS of the SF-12 v.1 at day 28. The means of the SF-12 v.1 PCS scores, derived from the returned questionnaires, will be compared in the ITI population between the two imaging groups. We will calculate the difference in means and the corresponding 95% confidence interval (CI).

We will test the non-inferiority hypothesis, excluding a 1-point difference or larger in favor of the chest X-ray strategy, using the one-sided 95% lower confidence limit of the difference between the mean SF-12 v.1 PCS scores.

The analysis will first be done using all available data. Then we will repeat the analysis after multiple imputation of the missing outcomes. Additionally, multivariable linear regression with imaging modality as a predictor as well as baseline characteristics (age, gender, comorbidity, presenting symptoms, clinical question on radiology application form) will be performed to further reduce confounding and to obtain a more precise estimate of the difference in means.

If the assumptions of normality are not met, we will calculate *P* values and 95% CI based on bootstrap resampling.

#### Analysis of benefit

Using a counterfactual framework, we will use multivariable modeling in an exploratory analysis of identifiable between-patient variability in effectiveness (difference in SF-12 v.1 PCS scores between the two strategies) as a function of measured baseline variables (age, gender, comorbidity, presenting symptoms, clinical question on radiology application form)*.*

#### Assessment and analysis of secondary outcomes

The assessment and analysis of the secondary outcomes will be discussed, separately for each secondary outcome, in the following paragraphs.

##### Mental health

Mental health is assessed by the MCS of the SF-12 v.1 questionnaire. The difference in mean MCS scores for the two imaging groups will be reported along with its 95% CI.

##### Length of hospital stay

The number of days spent in the clinical ward and/or intensive care unit (ICU) of the Amsterdam UMC, location AMC and SG were recorded in the electronic case report forms (eCRFs). If patients were transferred to another hospital, the number of days spent in the clinical ward or ICU at that hospital was recorded. Length of hospital stay (LOS) is defined as the number of days between admission and discharge. Day of admission and day of discharge count as one hospital day.

LOS will be analyzed by comparing the medians between the two groups and determining the difference with a 95% CI, based on the Hodges-Lehmann estimator.

##### Mortality within 28 days

The date of admission and, when applicable, date of death within 28 days were recorded in the eCRF. Date of death was extracted from the hospital electronic patient record. Firstly, mortality rate will be expressed as an absolute risk difference with 95% CI. In addition, we will perform a time-to-event analysis using proportional hazards modeling.

##### Quality-adjusted life years (QALYs) during the first 28 days (based on EQ-5D-5L health status scoring profiles)

The EQ-5D-5L questionnaire is administered at day 28 to score a patient’s health status, which is subsequently transposed into QALYs by applying an existing Dutch time trade-off-based health utility algorithm for the EQ-5D-5L and dividing the resulting health utility values by 13.04 (365.25/28) [23].

The difference in mean QALYs along with its 95% CI following non-parametric bootstrapping will be reported.

##### Diagnosis at ED discharge as compared to the final post hoc diagnosis at day 28

ED discharge diagnoses (day 0 diagnoses), as concluded by the attending physician at the ED, will be derived from the electronic health record by reviewers. To avoid personal interpretation of the ED discharge diagnoses and to facilitate uniformity among reviewers, a guideline for interpretation of the ED discharge diagnoses has been made. Each patient will be reviewed independently by two reviewers. If they agree on the diagnosis, this diagnosis will be assigned accordingly. In case of disagreement, a third reviewer (a physician with at least 1 year of clinical experience) will independently evaluate the patient as well, after which the third observer will be deblinded to the diagnoses of the first two reviewers. A final diagnosis will be assigned by the third reviewer after taking all reviews into account. The use of this method has been tested in two pilot studies.

The final post hoc diagnosis considers all available clinical, radiological, and microbiological data at 28 days of follow-up. Methods are described elsewhere [24]. In brief, a diagnostic handbook has been developed for the OPTIMACT study to define these post hoc diagnoses, enabling standardized and reproducible categorization. The handbook describes diagnostic reference standards for frequently occurring non-traumatic thoracic diseases like influenza, pneumonia, cardiac failure, etc. Each patient will be reviewed independently by two reviewers using the diagnostic handbook. In case of disagreement, a third reviewer (a physician with at least 1 year of clinical experience) will independently evaluate the patient as well. If the three reviewers still disagree after the case has been discussed in a plenary discussion, or if the diagnostic handbook indicates referring the case because of complexity, a final diagnosis will be made by an independent adjudication committee consisting of specialists in internal medicine, pulmonology, cardiology, and chest radiology. The members of the adjudication committee should not have been involved in the care of the study patients at the Amsterdam UMC, location AMC or SG during the study period. The efficiency and validation of this post hoc day 28 methodology, serving as the reference standard by the assessment of interobserver agreement, will be reported separately.

We will calculate the diagnostic accuracy of the chest X-ray and ULD chest CT by comparing the day 0 diagnosis at ED discharge with the post hoc diagnosis at day 28, using the latter as the clinical reference standard. This will be expressed as proportions of correct diagnosis with 95% CI. We will use the chi-square test statistic to compare the proportions of correct diagnoses between both groups.

##### Number of patients in follow-up because of incidental findings on chest X-ray or ULD chest CT

We will report the absolute difference in proportions between the two groups, with 95% CI.

##### Health care costs

Other uses of health care resources as well as the costs of care, loss of work productivity, and out-of-pocket expenses are reported as means per imaging group with their differences reported along with 95% CIs based on (non-parametric) bootstrapping. These secondary outcomes are fully addressed and separately reported from the clinical outcomes.

## Discussion

The aim of the OPTIMACT study is to evaluate the effectiveness of replacing chest X-ray with ULD chest CT on patient outcomes in the diagnostic work-up of patients suspected of having non-traumatic pulmonary disease at the ED. This SAP is written to prepare the main analyses that will be conducted after the data are available, and to increase transparency, allowing others to comment on our plans.

In 2011, dyspnea was the chief complaint in 3.7 million visits (2.7%) of more than 136 million visits to US EDs. Dyspnea-related pulmonary complaints (cough, chest discomfort) contributed to 8.2% of ED visits [25]. A chest X-ray is a standard diagnostic procedure in these patients thought to have non-traumatic pulmonary disease. The chest X-ray helps to elucidate important causes for pulmonary complaints, such as pneumonia, congestion, and pneumothorax, at a very low ionizing radiation dose (0.05 mSv). CT is a more accurate technique, but because of the much higher radiation dose (5 mSv), a standard chest CT scan is not suitable for routine imaging in dyspneic patients [26, 27]. The new CT scanners are also capable of acquiring ULD chest CT. The image quality of a ULD chest CT is less than that of a standard chest CT, but ULD chest CT gives a high level of diagnostic confidence in patients suspected of pulmonary disease at the ED [28]. Therefore, ULD chest CT may lead to more timely diagnoses, more timely treatment, and improved patient management compared to standard chest X-ray.

After this trial we will have precise estimates of the effectiveness of replacing chest X-ray with ULD chest CT, in terms of health outcomes, measured by a generic health-related quality-of-life instrument. We anticipate that ULD chest CT will have a small beneficial effect, allowing us to exclude a negative effect on health outcomes. We will also have precise estimates of the cost consequences of replacing chest X-ray with ULD chest CT, starting with the changes in immediate imaging costs and the costs of patient management, guided by the imaging results.

## Data Availability

The datasets generated and/or analyzed during the current study are not publicly accessible but are available from the corresponding author on reasonable request.

## Abbreviations

AMC: Academic Medical Center
Amsterdam UMC: Amsterdam University Medical Centers
CI: Confidence interval
CONSORT: Consolidated Standards of Reporting Trials
CT: Computed tomography
eCRF: Electronic case report form
ED: Emergency department
EQ-5D-5L: EuroQol five-dimension, five-level
IC: Informed consent
iMCQ: iMTA Medical Consumption Questionnaire
iMTA: Institute for Medical Technology Assessment
iPCQ: iMTA Productivity Cost Questionnaire
IPP: Imaging-per-protocol
ITI: Intention-to-imaging
LOS: Length of hospital stay
MCS: Mental component summary
PCS: Physical component summary
QALY: Quality-adjusted life year
RCT: Randomized controlled trial
SAP: Statistical analysis plan
SD: Standard deviation
SF-12: Short Form-12
SG: Spaarne Gasthuis
STARD: Standards for Reporting of Diagnostic accuracy studies
ULD chest CT: Ultra-low-dose chest computed tomography
